# Medical/Pharmacy Students Interprofessional Older Adult Medication Interactions/Contraindications Project

**DOI:** 10.1093/geroni/igab046.3017

**Published:** 2021-12-17

**Authors:** Marilyn Gugliucci, Victoria Thieme

**Affiliations:** 1 University of New England College of Osteopathic Medicine, Kennebunk, Maine, United States; 2 Univesity of New England College of Osteopathic Medicine, University of New England, Maine, United States

## Abstract

The University of New England College of Osteopathic Medicine (UNECOM) Geriatrics Education Mentors [GEM] program, established in 2014, pairs UNECOM students with older community living adults. GEM assignments focus on health review, medical humanities, and geriatrics training. Each year approximately 90 older adults participate in GEMs. In 2019, the GEM program was expanded with Geriatrics Workforce Enhancement Program (GWEP) grant funding to: include first year medical students, include 2 additional assignments (4 assignments over 10 months to 6 assignments over 18 months), and to create interprofessional student collaboration. In the new GEM Assignment 4: Medication Interactions/Contraindications, UNECOM students with their GEM compiled details on the GEM’s medication list (prescriptions, herbal, OTC); one of 4 Ms of Age Friendly Health Care. UNECOM students (84 pairs) were then assigned to UNE School of Pharmacy (SOP) students (42 SOP students had 2 UNECOM pairs) to conduct a “Lexicomp” (App) medication interactions and Beers Criteria review. UNECOM students documented findings with the SOP student partner; discussed the processes of review with their GEM and the resultant findings; documented the GEM’s questions and how the UNECOM student answered those questions; and discussed next steps for the GEM regarding options for different medications - especially follow up with their prescribing physician(s) for any noted interactions/contraindications. For GEMs with few medications, a mock medication list was assigned to ensure student experiences with medication reviews and GEM discussion. Although time intensive preparation is required, UNECOM & SOP students attained significant learning as did the GEM mentors.

